# Uncovering the Role of Thrombospodin-1 and Occludin as Potential Prognostic and Diagnostic Biomarkers in Traumatic Brain Injury

**DOI:** 10.3390/ijms27020571

**Published:** 2026-01-06

**Authors:** Céline Decouty-Pérez, Inés Valencia, María Alvarez-Rubal, Elena Martínez-Cuevas, Víctor Farré-Alins, María J. Calzada, Anna Penalba, Joan Montaner, Javier Rodríguez de Cía, Mario Taravilla-Loma, Borja J. Hernández-García, Esther Fuertes-Yebra, Águeda González-Rodríguez, Ana Belen Lopez-Rodriguez, Javier Egea

**Affiliations:** 1 Molecular Neuroinflammation and Neuronal Plasticity Research Laboratory, Instituto de Investigación Sanitaria-Princesa IIS-IP, Hospital Universitario Santa Cristina, 28009 Madrid, Spain; celinedecouty96@gmail.com (C.D.-P.); valenciafernandezines@gmail.com (I.V.); maria.alvarezrubal@gmail.com (M.A.-R.); martinezcuevas.elena@gmail.com (E.M.-C.); victorfarre@hotmail.com (V.F.-A.); javier.rodriguezde@salud.madrid.org (J.R.d.C.); esther.fuertes@salud.madrid.org (E.F.-Y.); 2 Department of Medicine, School of Medicine, Universidad Autónoma of Madrid, 28029 Madrid, Spain; mariajose.calzada@uam.es; 3 Instituto Investigación Sanitaria-Princesa IIS-IP, 28006 Madrid, Spain; 4 Neurovascular Research Laboratory, Vall d’Hebron Institute of Research (VHIR), Universitat Autònoma de Barcelona, 08035 Barcelona, Spain; anna.penalba@vhir.org (A.P.); jmontaner-ibis@us.es (J.M.); 5 Department of Neurology, Hospital Universitario Virgen Macarena, 41004 Sevilla, Spain; 6 Service of Neurosurgery, Hospital Universitario La Paz, 28029 Madrid, Spain; mtaravillaloma@gmail.com (M.T.-L.); borjajhega@gmail.com (B.J.H.-G.); 7 Instituto de Investigaciones Biomédicas Sols-Morreale (Centro Mixto CSIC-UAM), 28029 Madrid, Spain; aguedagr.phd@gmail.com; 8 Centro de Investigación Biomédica en Red de Diabetes y Enfermedades Metabólicas Asociadas (CIBERDEM), 28029 Madrid, Spain

**Keywords:** traumatic brain injury, biomarkers, blood–brain barrier, thrombospondin-1, tight junction’s protein

## Abstract

Traumatic brain injury (TBI) is a highly heterogeneous disease and achieving an accurate diagnosis remains a significant challenge. Biomarkers play a crucial role in minimizing the reliance on invasive techniques like computed tomography, which also have significant economic costs. Human samples were obtained from prospective cohort studies. Mice were subjected to an experimental model of traumatic brain injury. Biomarker levels, gene expression, and blood–brain barrier integrity were analyzed using ELISA, qRT-PCR, and Evans Blue assay; data were statistically evaluated using parametric or non-parametric tests as appropriate. This study focuses on evaluating the role of matricellular protein thrombospondin-1 (TSP-1) and the tight junction proteins occludin and ZO-1 as potential biomarkers of TBI. We showed that lower serum TSP-1 levels correlated with poor patient outcomes at 6 months compared to those patients with a good outcome. Additionally, the disruption of the blood–brain barrier (BBB) and subsequent release of tight junction proteins allowed us to identify occludin as a potential biomarker for prognosis in a cohort of TBI patients and as a diagnosis biomarker in a subgroup of patients with mild TBI, but its discriminative power as a diagnosis biomarker appears modest, as reflected by an AUC of 0.693. On the other hand, ZO-1 exhibited increased levels but limited diagnostic utility. These findings highlight the critical role of TSP-1 in maintaining BBB integrity and regulating the inflammatory response after a TBI, supported by the worsened condition observed in TSP-1-deficient animals. These results demonstrate the potential of TSP-1 and occludin as valuable biomarkers for secondary injury and disease progression in patients with mild to moderate/severe TBI.

## 1. Introduction

Traumatic brain injury (TBI) is one of the leading causes of disability and even death worldwide, commonly resulting from traffic accidents, sports injuries, violence in combat zones, and falls. TBI causes neurological damage regardless of age, sex, or genetic background, and the survivors of a TBI often experience lasting behavioral, cognitive, and emotional changes [1]. TBI is a complex process that involves a cascade of molecular and cellular events, leading to both immediate and delayed brain damage. Primary injury occurs immediately after the impact, while the secondary injury response develops over time, aggravating tissue damage and functional impairment. This second response includes neuroinflammation, excitotoxicity, oxidative stress, mitochondrial dysfunction, and impaired cerebral blood flow. In this context, dysfunction of the vascular system plays an important role in the progression of a TBI, contributing to hemorrhage, edema, and blood–brain barrier (BBB) disruption [2]. Several studies using postmortem human tissue or mouse models have shown that the integrity of the BBB remains compromised months and even years after a TBI [3,4]. A more comprehensive understanding of vascular repair mechanisms is needed, as it may lead to the development of new angiogenic therapies, and the development of biomarkers related to BBB status [5]. TBI is classified from a variety of perspectives, including the classic severity score (Glasgow Coma Scale, GCS) which categorizes injuries as mild, moderate, or severe depending on the level of altered consciousness [6]. Clinicians commonly use the GCS alongside symptoms like headache, nausea, vomiting, loss of consciousness, and amnesia to evaluate TBI patients and determine their risk for intracranial complications. Computed tomography (CT) scans are frequently used to detect and evaluate trauma-related brain lesions; however, they are expensive, involve radiation exposure, and only around 5% of cases reveal detectable brain damage [6]. This emphasizes the importance of establishing clear criteria to determine whether a patient requires a CT scan. For this reason, many efforts have been made in the last decades to develop novel blood biomarkers to predict and monitor brain injury progression [7] and reliably differentiate between CT-positive and CT-negative patients [8]. Although current consensus guidelines support the use of GFAP, UCH-L1, and S100B for acute and subacute diagnosis and the classification of TBIs, the working group also highlights emerging evidence that biomarkers such as NfL or phosphorylated-Tau may provide prognostic information at chronic timepoints (>30 days), underscoring the need for further investigation of additional candidate markers [9].

The BBB is composed of different cell types including endothelial cells, pericytes, and astrocytes. Following primary injury, its permeability increases, contributing to brain edema. While part of this damage is irreversible due to endothelial cell death, dysfunction of endothelial tight junction proteins can be repaired [10,11]. Occludin and ZO-1 are key tight junction proteins that regulate paracellular permeability and maintain BBB integrity. Occludin contributes to barrier stability, while ZO-1 links transmembrane proteins to the cytoskeleton, coordinating junction assembly [12]. All these processes contribute to the extravasation of peripheral immune system cells leading to increased inflammation in the injured area. Previous studies on brain injury models have suggested that tight junction proteins may be potential biomarkers of neonatal hypoxic/ischemic brain injury [13], Alzheimer’s disease [14], and multiple sclerosis [15].

Thrombospondin-1 (TSP-1) is a matricellular glycoprotein that plays a crucial role in modulating cell–cell and cell–matrix interactions in different physiological processes, which include platelet aggregation, angiogenesis, synaptogenesis, dendritic cell maturation, and wound healing [16]. Following TBI, TSP-1 is implicated in three key processes: (i) BBB integrity, (ii) vascular remodeling, and (iii) synaptogenesis [17].

Therefore, the aim of this study was to evaluate and compare the potential of TSP-1 and tight junction proteins (occludin and ZO-1) as diagnostic and prognostic biomarkers in the serum of TBI patients. Additionally, we aimed to explore the possible relationship of TSP-1, BBB disruption, and tight junction proteins in the context of TBI, using the closed-head injury TBI mouse model.

## 2. Results

### 2.1. Serum Profile of TSP-1, Occludin, and ZO-1 and Their Correlation with Prognosis

**Figure 1:**
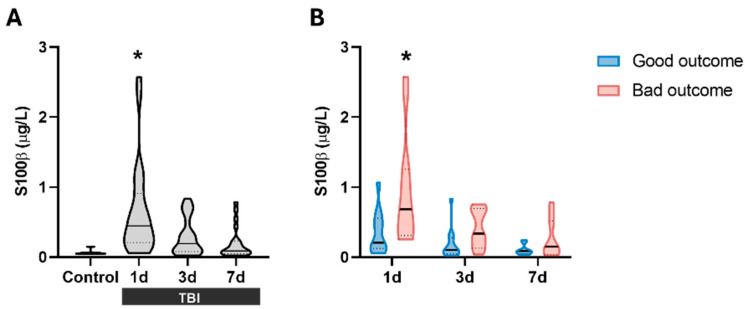
S100β levels in the serum of patients. (**A**) S100β significantly increases at 1 day after TBI (n = 23) compared to control subjects, but not at 3 days (n = 23) or 7 days (n = 21) compared to control subjects (control group, n = 7). * *p* < 0.05 vs. Control, using Kruskal–Wallis analysis with Dunn’s post-hoc test. (**B**) S100β levels stratified by prognosis at 6 months based on GOSE scale. At 1 day post-TBI, S100β levels were significantly higher in patients with bad outcomes (n = 12) compared to those with good outcomes (n = 11). No significant differences were observed at later time points. * *p* < 0.05 vs. good outcome at the same time point, using Two-way ANOVA with Bonferroni’s post-hoc test. Data are presented as the median with the interquartile range.

Table 1 shows the demographic characteristics of TBI patients included in the first part of this study. To validate our cohort, S100β protein levels were analyzed at three time points after injury (1, 3, and 7 days) (Figure 1A). S100β serum levels showed a significant increase at 1 day (median = 0.446 µg/L; IQR = 0.208–0.912), followed by a gradual decline at 3 days (median = 0.193 µg/L; IQR = 0.078–0.523) and 7 days (median = 0.089 µg/L; IQR = 0.048–0.231). Subsequently, S100β data were classified according to the Glasgow Outcome Scale–Extended (GOSE) at 6 months to evaluate their correlation with prognosis. Figure 1B shows that patients with a poor prognosis at 6 months post-injury had significantly higher S100β1d values (median = 0.208 µg/L; IQR = 0.127–0.559) than patients with a good prognosis at 1 day after lesion. However, the protein values drastically decreased after the first day and no significant differences in prognosis were observed after 3 and 7 days. These data confirm that our patient cohort is representative of this brain damage.

**Figure 2:**
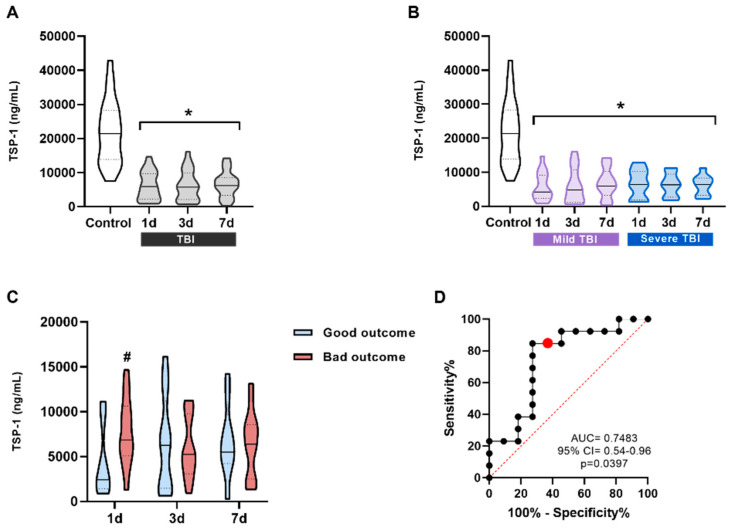
TSP-1 levels in the serum of patients and their correlation with prognosis. (**A**) TSP-1 levels in all patients compared to the control group. TSP-1 significantly decreases at all three measured times, 1 day (n = 24), 3 days (n = 24), and 7 days (n = 22) after a TBI compared to the control subjects (n = 24). (**B**) TSP-1 levels in patients classified into two groups according to the severity of their injury based on the GCS scale (mild and severe TBI). TSP-1 levels were always significantly lower and did not correlate with severity at any of the times. * *p* < 0.05 vs. Control, using One-way ANOVA followed by Bonferroni’s post-hoc test. (**C**) TSP-11d values were significantly higher in the poor prognosis group. # *p* < 0.05 vs. good prognosis. To see the effect of prognosis at different times, a *t*-test was performed between each time separately. (**D**) ROC curve for TSP-11d levels to predict possible differences between the good outcome group (n = 11) and the bad outcome group (n = 13). The red dot represents the value with the highest Youden index on the curve. Data are presented as the median with the interquartile range.

Serum TSP-1 levels decreased at 1, 3, and 7 days post-TBI, with no significant differences between time points: TSP-11 day (median = 5880 ng/mL; IQR = 2283–9675), 3 days (median = 5722 ng/mL; IQR = 2095–16,138), and 7 days (median = 6167 ng/mL; IQR = 3362–14,218) (Figure 2A). To evaluate whether TSP-1 could have diagnostic potential, patients were categorized based on their lesion severity. However, no significant differences were found between mild and severe lesions at any of the three time points (Figure 2B). Regarding prognosis, serum levels of TSP-11d were significantly higher in patients with a poor prognosis (median = 6865 ng/mL, IQR = 5067–10,622) than in patients with a good prognosis at 6 months (median = 2414 ng/mL, IQR = 1432–8767). However, it was not discriminatory at 3 and 7 days post-TBI (Figure 2C). ROC curve analysis was used to evaluate whether TSP-11d might distinguish between patients with good and poor prognoses. The area under the curve (AUC) showed an acceptable discrimination of this protein 1 day post-TBI (AUC = 0.745, 95% CI = 0.54–0.96, *p* = 0.039) (Figure 2D).

**Figure 3:**
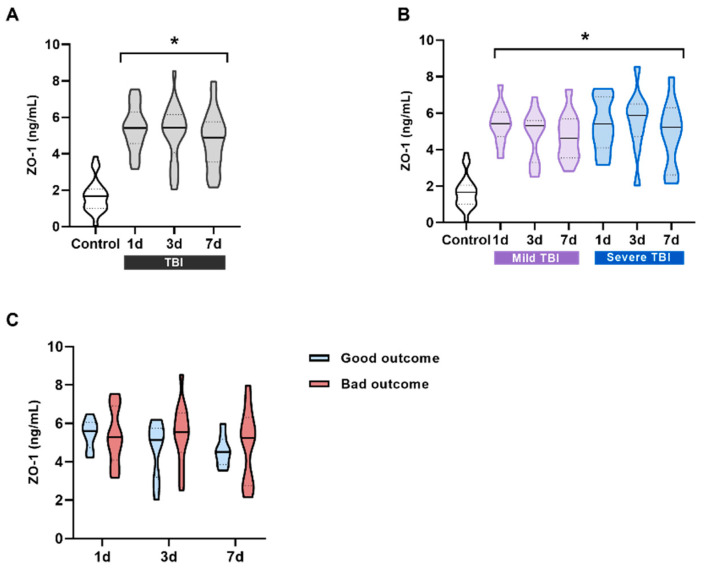
ZO-1 levels in the serum of patients and their correlation with prognosis. (**A**) ZO-1 serum levels in all patients compared to the control group. ZO-1 significantly increases at all three measured times, 1 day (n = 21), 3 days (n = 23), and 7 days (n = 22) after a TBI compared to the control subjects (n = 23). * *p* < 0.05 vs. Control, using One-way ANOVA followed by Bonferroni’s post-hoc test. (**B**) Patients were classified into two groups according to the severity of their injury based on the GCS (mild and severe TBI). ZO-1 levels were always significantly higher and did not correlate with severity at any of the times. * *p* < 0.05 vs. Control, using One-way ANOVA followed by Bonferroni’s post-hoc test. (**C**) No significant differences were observed between ZO-1 values at different times for patients with good/bad outcomes. Data are presented as the median with the interquartile range.

Considering the critical role of BBB disruption in the pathophysiology of TBI and its association with TSP-1, we next measured the levels of the tight junction proteins ZO-1 and occludin in the serum of these patients. ZO-1 levels were significantly increased in all TBI patients compared to the controls (Figure 3A). However, when patients were stratified according to their injury severity, no significant differences were observed between mild and severe cases (Figure 3B). Similarly, serum ZO-1 levels showed no significant differences between groups when analyzed in relation to patient outcome (Figure 3C). These findings suggest that although ZO-1 levels are markedly elevated following a TBI, they do not serve as a reliable biomarker for either diagnosis or prognosis in these patients.

**Figure 4:**
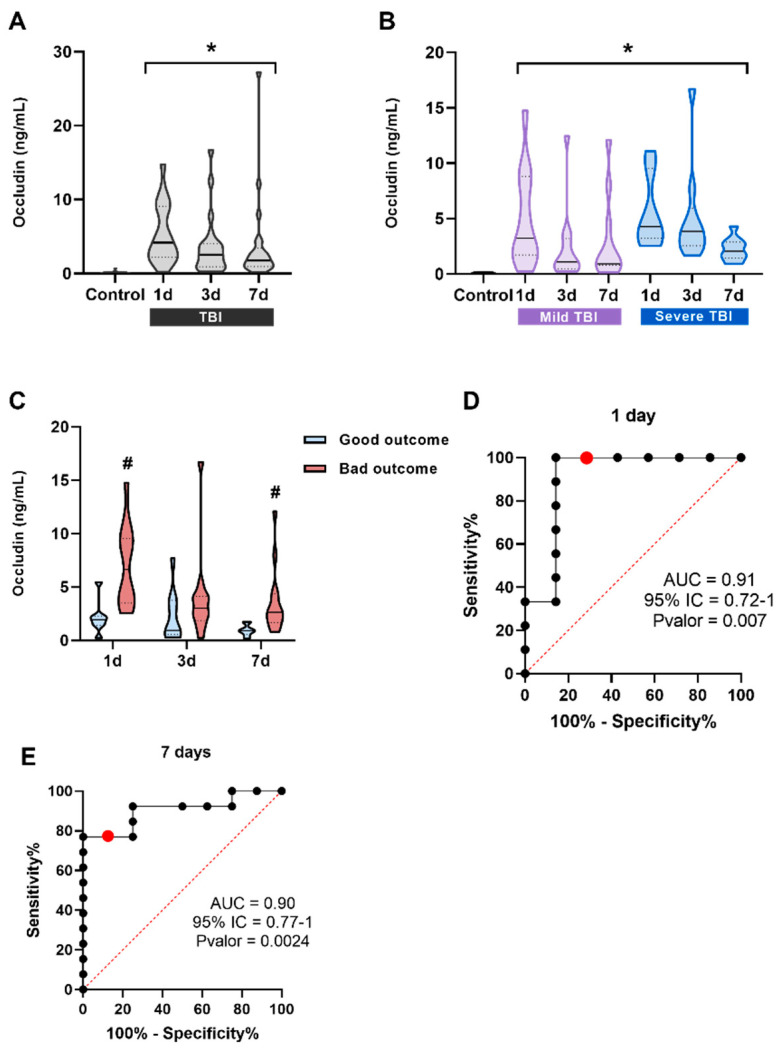
Occludin levels in the serum of patients and their correlation with prognosis. (**A**) Occludin significantly increases at all three measured times, 1 day (n = 19), 3 days (n = 22), and 7 days (n = 22) after a TBI compared to the control subjects (n = 23). * *p* < 0.05 vs. Control, using Kruskal–Wallis analysis with Dunn’s post-hoc test. (**B**) Patients were classified into two groups according to the severity of their injury based on the GCS (mild and severe TBI). Occludin levels were always significantly lower and did not correlate with severity at any of the times. * *p* < 0.05 vs. Control, using Kruskal–Wallis analysis with Dunn’s post-hoc test. (**C**) Occludin1d and occludin7d levels were significantly higher in the bad outcome group (1 day, n = 12 and 7 days, n = 13) compared to the good outcome group (1 day, n = 7 and 7 days, n = 8) at the same time points. # *p* < 0.05 vs. good outcome. To assess the effect of prognosis at different times, a *t*-test was performed between each time point separately. (**D**) ROC curve analysis was used for occludin1d levels to predict possible differences between the good and the bad outcome groups. (**E**) ROC curve analysis was used for occludin7d levels to predict possible differences between the good and the bad outcome groups. The red dot represents the value with the highest Youden index on the curve. Data are presented as the median with the interquartile range.

Occludin was chosen as the second tight junction protein selected for evaluation because, unlike ZO-1, it is highly concentrated in brain endothelial cells and plays a crucial role in vascular permeability [18]. TBI patients had significantly higher occludin levels compared to controls at all time points: occludin1d (median = 4.182 ng/mL; IQR = 2.186–9.109), occludin3d (median = 2.542 ng/mL, IQR = 0.917–4.006), and occludin7d (median = 1.774 ng/mL, IQR = 0.9288–3.432) (Figure 4A). Then, patients were stratified based on GCS (mild and severe), but there were no differences between the groups, suggesting that there is no relationship between TBI severity and serum occludin levels (Figure 4B). Finally, we investigated the correlation between serum occludin levels and patient prognosis for 6 months post-TBI. Significant differences were found in occludin levels between patients with good and poor outcomes according to the GOSE at 1 and 7 days after a TBI (Figure 4C). The AUCs of ROC curves for occludin1d and occludin7d indicated that this protein has highly promising potential as a biomarker, as it demonstrated nearly perfect discrimination for predicting the potential prognosis of patients with mild to severe TBIs at 6 months post-injury at both time points (AUC_1d_ = 0.9286, 95% CI = 0.788–1; AUC_7d_ = 0.9038, 95% CI = 0.771–1) (Figure 4D,E).

Finally, the possibility of combining biomarkers to improve predictive capacity was explored. Despite occludin showing the strongest potential as a biomarker, combining it with TSP-1 resulted in a loss of significance. Additionally, S100β, a biomarker known to improve predictive capacity when combined with other proteins [19], was also considered. However, in our patient cohort, none of the proteins combined with S100β showed a significant improvement in predictive capacity compared to their individual evaluation.

### 2.2. Expression Profile of TSP-1 and Occludin and Their Correlation with Prognosis in a Subgroup of Patients with Mild TBIs

After verifying the potential role of TSP-1 and occludin in the prognosis of mild and severe TBIs, we focused on analyzing a subgroup of patients with significant clinical relevance. The cohort consisted of 84 patients with mild TBIs and 23 control subjects. Diagnosing a mild TBI can be challenging, as a CT scan is often required to confirm brain injury. However, CT imaging is costly and exposes patients to radiation, so finding blood biomarkers to differentiate brain injuries is of the utmost importance. The objective was to evaluate whether the serum levels of TSP-1 and occludin 1 day post-TBI could correlate with CT scan findings in these patients, evaluating their potential as diagnostic biomarkers.

**Figure 5:**
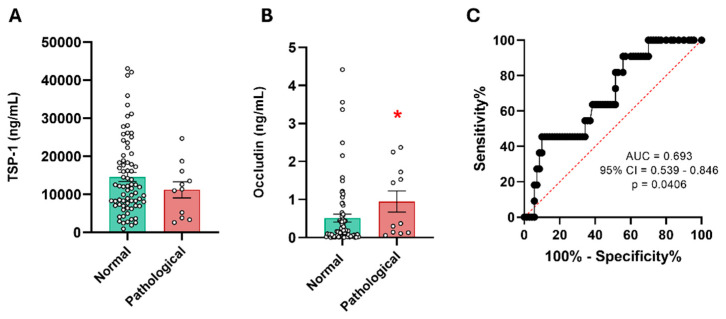
Serum levels of TSP-1 and occludin in patients with mild TBIs and their correlation with CT. (**A**) The serum levels of TSP-1 in patients with mild TBIs. No significant differences were found in the TSP-11d levels of patients with mild TBIs dichotomized according to their CT results (normal n = 73 and pathological n = 11). (**B**) The serum levels of occludin in patients with mild TBIs showed a significant increase in those with abnormal CT results. * *p* < 0.05 vs. Normal CT group, using Student’s *t*-test. (**C**) ROC curve analysis was used for occludin1d levels to predict possible differences between the normal CT group (n = 70) and the abnormal CT group (n = 11). CT: Computed tomography.

Table 2 shows the demographic characteristics of this patient cohort. TSP-1 showed no significant differences between the groups of patients with normal CT scans (median = 12,017 ng/mL, IQR = 7205–19,542 ng/mL) and pathological CT scans (median = 11,336 ng/mL, IQR = 3822–16,120 ng/mL). These results suggest that TSP-1 is not effective in distinguishing CT outcomes in patients with mild TBIs (Figure 5A).

We then investigated the potential predictive value of occludin in determining the need for a CT scan in these patients. The analysis revealed significant differences in serum occludin levels between the two groups of patients (normal CT vs. pathological CT) (Figure 5B). The median occludin level in the normal CT group was 0.155 ng/mL (IQR = 0.035–0.570), while in the pathological CT group it was 0.367 ng/mL (IQR = 0.129–1.722). Additionally, a ROC curve analysis was performed to evaluate the discriminatory ability of serum occludin levels. The AUC was 0.693, which did not reach the necessary values to be considered a good discriminator (Figure 5C).

### 2.3. TSP-1 Deficiency Worsens the Neuroinflammatory Response After TBI

**Figure 6:**
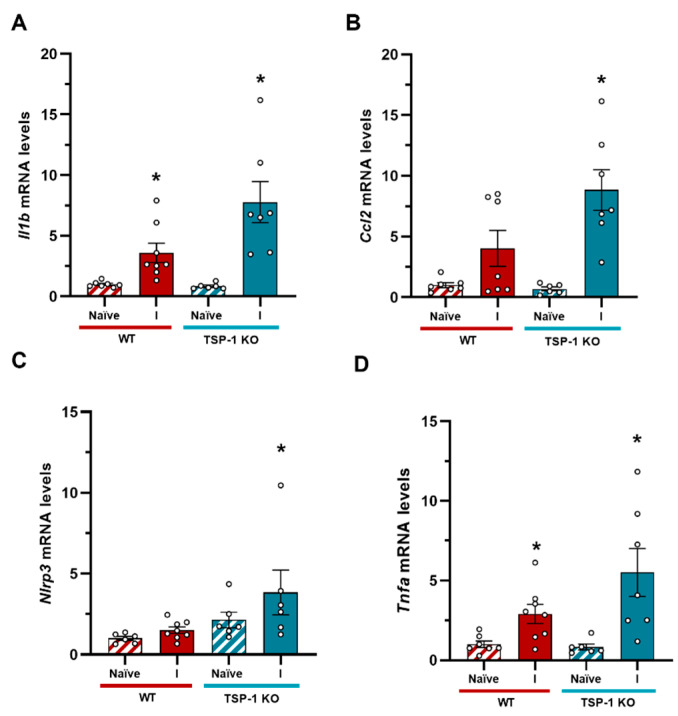
Inflammatory profile of TSP-1 KO animals after TBIs. (**A**) Il1b expression levels were significantly elevated in the ipsilateral hemisphere in the WT mice, and this expression significantly increased in the TSP-1 KO animals after injury. (**B**) Ccl2 expression was exacerbated in the ipsilateral side of the TSP-1 KO mice compared to the WT animals. (**C**) Nlrp3 levels were only significantly higher in the TSP-1 KO animals after a TBI. (**D**) An increase in Tnfa expression was observed in both the WT and TSP-1 KO animals after a TBI compared to the naïve animals. Mean ± SEM (n = 6–8). * *p* < 0.05 vs. naïve of their genotype, using Kruskal–Wallis analysis followed by Dunn’s post-hoc test. A *t*-test was performed to assess the effect of genotype. (WT: Wild type; KO: knock out; I: ipsilateral).

To further investigate the potential role of TSP-1 in the context of TBI, we conducted a series of experiments using a moderate/severe closed-head injury TBI animal model. First, we investigate the effect of TSP-1 deficiency on the regulation of neuroinflammatory processes occurring after the TBI. The induced TBI resulted in a significant increase in Il1b mRNA levels in both the WT and TSP-1 KO animals, with an exaggerated response in the KO animals (Figure 6A). The mRNA levels of the monocyte chemoattractant protein C-C motif chemokine ligand 2 (Ccl2) were two-fold higher in the ipsilateral hemisphere of the TSP-1 KO animals compared to the WT animals (Figure 6B). The expression of Tnfa, which is primarily secreted by glial cells such as astrocytes and microglia, was also increased at the mRNA level in both the WT and TSP-1 KO animals after the TBIs, with a significant higher response in the TSP-1 KO animals (Figure 6D). These results demonstrate the role of TSP-1 in regulating the inflammatory response following a TBI.

### 2.4. TSP-1 Deficiency Aggravates BBB Disruption and Diminishes Angiogenic Repair After TBI

**Figure 7:**
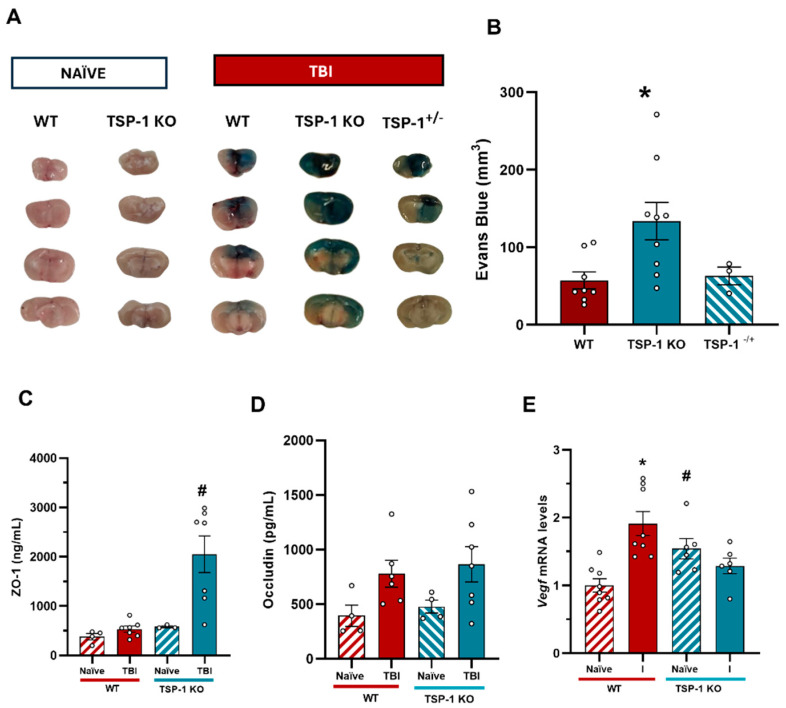
Effects of TSP-1 deficiency on BBB integrity and angiogenic response. (**A**) Representative images of 2 mm brain slices for the quantification of Evans Blue. (**B**) Quantification of Evans Blue extravasation. (n = 3–9) Mean ± SEM. One-way ANOVA was performed followed by Bonferroni’s post-hoc test *p* ≤ 0.05. (* vs. WT). (**C**) ZO-1 serum levels did not change 24 h post-TBI in the WT animals while in the TSP-1-/- mice they were significantly increased. (**D**) Occludin levels were significantly elevated in the injured animals 24 h post-TBI regardless of their genotype. Mean ± SEM (WT naïve n = 4, WT TBI n = 7–8, TSP-1 KO naïve n = 3–4, TSP-1 KO TBI n = 7)). Kruskal–Wallis analysis was performed followed by Dunn’s post-hoc test. *p* ≤ 0.05. (* vs. naïve of their genotype, # vs. WT). (**E**) Vegf mRNA levels increased in the ipsilateral hemisphere of the injured WT animals. An increase in Vegf was observed in the naïve TSP-1 KO animals compared to the naïve WT mice; yet, the injuries did not modify the Vegf mRNA levels in the TSP-1 KO animals. * *p* < 0.05 vs. naïve of their genotype, and # *p* < 0.05 vs. WT, using Two-way ANOVA with Bonferroni’s post-hoc test.

To further understand the role of TSP-1 in maintaining BBB integrity following a TBI, we conducted BBB integrity measurements in WT and TSP-1 KO animals. Figure 7A shows that the TSP-1-deficient animals exhibited significantly higher Evans Blue extravasation than the WT animals 24 h after the TBI, suggesting a greater BBB disruption. Furthermore, BBB integrity was also analyzed in heterozygous animals (TSP-1^−/+^), demonstrating that the presence of a single allele is sufficient to restore the WT phenotype in terms of Evans Blue extravasation (Figure 7B).

To confirm this loss of BBB integrity, the two tight junction proteins previously analyzed in patient samples (ZO-1 and occludin) were measured. Serum ZO-1 levels were significantly elevated in the TSP-1 KO animals 24 h post-TBI compared to the WT animals (Figure 7C). In contrast, the serum levels of occludin significantly increased after the injuries regardless of genotype (Figure 7D). Furthermore, we measured Vegf gene levels, a molecule involved in post-TBI vascular regeneration, which showed a significant increase in the ipsilateral side, with no differences between the naïve animals and the contralateral side (Figure 7E). These results support the notion that TSP-1 deficiency contributes to a differential vascular and endothelial permeability structure within the BBB, compromising its integrity following a TBI. Further investigation into the precise neurovascular architecture of TSP-1 KO mice will provide deeper insight into the mechanisms underlying BBB dysfunction and the associated exacerbation of neuroinflammation after TBI.

## 3. Discussion

TBI is unpredictable, and brain damage is not always easily detectable by medical professionals and procedures. In adults, cognitive impairment and symptoms occur during the acute phase of TBI, but it typically takes 3 to 12 months to resolve [20]. Due to uncertainties in patient prognosis and monitoring, the interest in biomarker research has increased in recent decades [7]. Biomarkers have shown promise in the early stages of TBI and have gained attention from a therapeutic perspective, suggesting that modulating specific biomarkers may open new research opportunities in the treatment of TBI patients [21]. The present work focuses on studying different biomarkers related to BBB integrity and angiogenesis. We identified occludin and TSP-1 as promising biomarkers for TBI diagnosis and prognosis in two different patient cohorts. Furthermore, we demonstrated that the TSP-1 protein plays a key role in maintaining BBB integrity, as its absence worsens Evans Blue extravasation and animal outcomes in relation to neuroinflammation and angiogenic repair after a TBI. A major challenge in biomarker validation has been the focus on a single sampling time point instead of evaluating time-dependent changes [22]. In our first cohort, samples were collected on days 1, 3, and 7 after injury, offering us an advantage over other single time point studies. In this study, S100β was detected at all time points evaluated, yet it was only significantly increased 1 day after a TBI (Figure 1A). These results are in line with a study of 154 patients that showed that the maximum release of this protein occurs approximately 27 h after injury [23]. Regarding the prognosis, we also observed significant differences in serum S100β1d levels when patients were classified according to their 6-month outcome (Figure 1B), suggesting that our cohort is representative of this pathology.

The first new protein examined as a potential biomarker was TSP-1, chosen for its role in regulating important processes such as angiogenesis, inflammatory responses, and tissue repair [17]. Previous work from our group demonstrated that TLR4 activation following a TBI increases TSP-1 release, which contributes to synaptic recovery days after the injury [24]. Our results showed a statistically significant decrease in TSP-1 levels in TBI patients at all time points compared to the controls, regardless of the measurement time or injury severity. However, when patients were stratified by prognosis (GOSE at 6 months), TSP-1 levels at day 1 post-injury were significantly higher in the patients with poor outcomes than in those with good outcomes (Figure 2C). The AUC of the ROC curve was 0.7483, suggesting an acceptable discrimination ability. These findings suggest that TSP-1 could serve as potential prognostic biomarker for TBI outcomes in clinical practice. These promising results need further validation in larger cohorts to confirm its biomarker potential.

An accurate diagnosis of TBI, as well as prognosis, is important to ensure proper medical care and avoid unnecessary radiation exposure from imaging tests such as a CT scan. At the same time, it prevents patients’ symptoms from being underestimated [25]. This issue is crucial in cases of mild TBIs, as subtle or nonspecific symptoms may appear. The first protein evaluated as a potential diagnostic biomarker to discriminate between patients with abnormal CT scans was TSP-1. These patients showed a slight trend toward reduced TSP-1 levels, but without statistical differences. These data suggest that TSP-1 does not correlate with CT scan results in mild TBI patients. TSP-1 is involved in brain repair and synaptogenesis after injury, so it may be associated with long-term effects rather than acute-phase lesions [26]. We analyzed occludin using the same approach as in the first cohort. The results showed that occludin could differentiate between patients with normal and abnormal CT scans, suggesting its potential as a diagnostic biomarker. The ROC curve showed acceptable discrimination (AUC = 0.693). As the mild TBI cohort included only a small number of patients with abnormal CT scans (approximately 10% of patients), these promising data need to be further explored in larger cohorts. Tight junction proteins such as ZO-1 and occludin play a crucial role in maintaining the integrity of the BBB by limiting diffusion between endothelial cells [27]. In healthy individuals, ZO-1 was detected in the blood, whereas occludin was barely detectable, indicating that its role is more brain-specific compared to ZO-1. In TBI patients, both proteins showed significantly higher levels at all time points compared to the controls. However, no significant differences were observed when patients were stratified by injury severity. When analyzing patients based on prognosis, serum occludin levels at 1 and 7 days after injury were higher in the patients with poor outcomes. The AUC of the ROC curves were 0.9286 for occludin1d and 0.9038 for occludin7d, indicating very good discrimination at both time points. Our results are consistent with those obtained in other brain injuries such as hypoxic/ischemic brain injury [28] and Alzheimer’s disease [29], in which the tight junction proteins claudin-V, occludin, and ZO-1 have demonstrated a role as promising biomarkers for cerebrovascular dysfunction. A key limitation of this study is the restricted statistical power, particularly in the analyses involving small prognosis or CT-based subgroups.

Choosing an animal model for TBI research is challenging due to the complexity and variability of a human traumatic brain injury and its underlying causes. To date, no model fully recapitulates the human biomechanical mechanisms of a TBI [30]. Our model was characterized by increased mRNA levels of proinflammatory cytokines, I1b and Tnfa, as well as the vascular growing factor Vegf in the affected area, confirming localized injury. The TSP-1 KO mice showed significantly higher Evans Blue extravasation than the WT animals 24 h after injury, suggesting that TSP-1 plays a critical role in maintaining BBB integrity, which is consistent with other TBI models [31]. To corroborate this protection that we are observing acutely, it would be very interesting to extend our model to longer time points, where other studies have shown tissue repair [32]. To further investigate the status of the BBB, we measured occludin and ZO-1 serum levels. While ZO-1 levels barely changed in the WT animals after injury, they remained significantly elevated in the TSP-1 KO mice, supporting the findings on BBB integrity. However, occludin levels remained elevated after TBI without significant genotypic differences. These findings, together with the BBB integrity experiments, highlight the role of TSP-1 in BBB permeability and correlate with the results obtained with patient serum. It is important to understand the connection between the BBB and inflammation. Moreover, recent work in intestinal inflammatory disorders has highlighted a tight bidirectional crosstalk between barrier integrity and inflammatory signaling, whereby a disruption of junctional proteins activates NF-κB, further amplifying inflammation and barrier breakdown; although described in the intestinal epithelium, this signaling axis is conserved and may be highly relevant to blood–brain barrier dysfunction after traumatic brain injury [33].

TSP-1 deficiency is associated with a proinflammatory gene profile, leading to an exaggerated response to injury when compared to WT animals. TSP-1 has both anti- and proinflammatory properties as it interacts with various receptors, suggesting a dual role in inflammation [17]. TSP-1 binding to CD36 and CD47 receptors inhibits inflammation by blocking leukocyte activation and adhesion to the endothelium. In vitro wound healing models have shown that TSP-1 deficiency prolongs inflammation by delaying wound closure, although it can also reduce transforming growth factor-β (TGF-β) activation, which is known for its anti-inflammatory and tissue repair effects [34]. On the other hand, when analyzing angiogenic and vascular repair markers after TBIs, we observed that Vegf mRNA levels increased in the injured WT animals, whereas in the TSP-1 KO animals, Vegf levels remained unchanged despite being inherently elevated at baseline (Figure 7E). Despite the exacerbated inflammatory response observed in these animals, this does not correlate with an additional VEGF increase (Figure 7E). This lack of VEGF upregulation in the TSP-1 KO mice is somewhat unexpected, as injury-induced angiogenesis is often accompanied by elevated VEGF levels [35]. TSP-1 is known to regulate VEGF signaling through multiple mechanisms, including inhibition via its receptors CD36 and CD47, which can block downstream VEGF signaling. Additionally, it has been described that TSP-1 can sequester VEGF directly, thereby modulating its availability and bioactivity [36,37]. However, in our model, VEGF levels remained unchanged, suggesting a more complex interaction or potential compensatory mechanisms that limit its expression. As Evans Blue extravasation was not increased in the naïve TSP-1 KO mice, we cannot assume that these animals have a basal increased BBB permeability. Instead, we hypothesize that their vascular structure and angiogenic repair capacity are inherently compromised, making them more susceptible to BBB disruption and enhanced secondary damage following TBI. The variation in VEGF and the role of TSP-1 may therefore influence and determine the progression of acute TBI responses, justifying its potential utility as a biomarker in this context, although further investigation is needed to understand the exact mechanism of TSP-1.

Despite the exacerbated inflammatory response observed in these animals, it does not correlate with an additional Vegf increase. The variation in Vegf and the role of TSP-1 may therefore influence and determine the progression of acute TBI response, justifying its potential utility as biomarker in this context, although further investigation is needed to understand the exact mechanism of TSP-1.

## 4. Materials and Methods

### 4.1. Human Samples

Study 1. The human samples were collected during an observational prospective cohort study carried out at the Department of Neurosurgery of Hospital Universitario La Paz (Madrid, Spain) between April 2017 and October 2018. Patients were included in accordance with the following criteria: aged between 18 and 85 years, hospital admission with a diagnosis of a closed-head injury, presentation within 24 h of injury, and admission to either the Intensive Care Unit or the Neurosurgery Ward. The exclusion criteria included a history of previous neurological disease or cognitive impairment, as well as the inability to undergo head CT, to provide a biological sample, or to complete a proper follow-up. Venous blood samples were obtained at 1, 3, and 7 days after suffering a TBI (PI-2153).

Study 2. The human samples were collected during an observational prospective cohort study at the Hospital de Vall D’Hebron (Barcelona, Spain) (PR(AG)195/2012 y PR(CS)27/2019). The samples were collected 24 h after the TBI and were stratified according to whether the CT scan imaging result was pathological or not.

### 4.2. Animals

C57Bl6/J (RRID: IMSR_JAX:000664) mice, approximately 3 months old (25–30 g), were obtained from the inhouse colony at the animal facilities of the Universidad Autónoma de Madrid (UAM, Madrid, Spain). TSP-1 KO (RRID: IMSR_JAX:006141) [38] mice were kindly provided by M. J. Calzada (Instituto de Investigación Biomédica del Hospital La Princesa, Madrid, Spain). The animals were housed in cages of four at 21 °C with a 12 h light/dark cycle and ingested water and food *ad libitum*. All animal experimentation was performed under the license PROEX 109/18 granted by the Ethics Committee of the Universidad Autónoma de Madrid (Madrid, Spain) and in compliance with the Cruelty to Animals Act, 1876, and the European Community Directive, 86/609/EEC. Every effort was made to minimize stress to the animals.

### 4.3. Traumatic Brain Injury Model

The mice were subjected to the closed-head injury (CHI) model in which, after being anesthetized with inhaled isoflurane, the head of each animal was immobilized and exposed to a free-fall 50 g weight dropped from a height of 34 cm. This procedure is adapted from Flierl et al., 2009 [39]. After the injury, the mice were closely monitored in an individual cage and oxygen was administered until regular breath was restored. This model produces severe TBIs, characterized by a strong inflammatory response and a 5–15% mortality rate. Group allocation was performed by a researcher blinded to the study outcomes and who generated the randomization sequence. The mice were randomly divided in 2 groups: naïve (no subjected to CHI, *n* = 8) and TBI (subjected to CHI, *n* = 8) for WT and TSP1 KO mice. The sample size was calculated considering the data of previous studies [24]. Animals that did not survive the first 24 h were excluded from analysis.

### 4.4. Blood–Brain Barrier Integrity Assessment

Evans Blue tracer (Sigma-Aldrich, Madrid, Spain) was diluted at 2% in saline and administered via intraperitoneal injection (3 mL/kg; i.p.) immediately after CHI. The animals were sacrificed 24 h after the trauma and their brains were gently extracted and sectioned in four 2 mm slices using a mouse brain slicer. Each slice was scanned, and the total area of Evans Blue extravasation was quantified using ImageJ 1.52e software (ImageJ software, National Institutes of Health, Stapleton, NY, USA). The results were represented as total extravasation volume (mm^3^).

### 4.5. Tissue Preparation

For transcriptional change analysis, the animals were terminally anesthetized 24 h after the TBI with a mix of ketamine–xylacine at a 1:2 ratio (Ketolar 50 mg/mL, Pfizer, NY, USA; Xilagesic 20 mg/mL, Calier Labs, Barcelona, Spain) and then rapidly, transcardially perfused with a 0.9% NaCl buffer. The brains were gently removed and a punch of the right (ipsilateral) and left (contralateral) hemispheres were snap frozen and stored at −80 °C until use. RNA and proteins were obtained by the double-extraction protocol with a Trizol reagent (TRI Reagent, Molecular Research Center, Inc., Cincinnati, OH, USA) according to the manufacturer’s instructions.

### 4.6. ELISA Assay

Human blood was used to determine the levels of S100β (Diasorin, Saluggia, Italy), TSP-1 (R&D Systems, Minneapolis, MN, USA), occludin (Elabscience, TX, USA), and ZO-1 (Elabscience, TX, USA) using a specific ELISA assay, following the manufacturer protocol. The mice were perfused with a 0.9% saline solution and blood was collected in tubes, which were kept at 4 °C overnight to allow the blood to clot. Afterward, the tubes were centrifuged at 1500 rpm for 10 min at 4 °C, and the serum was collected.

### 4.7. Quantitative Real-Time Polymerase Chain Reaction (qRT-PCR)

Following the extraction of RNA, the amount and quality of each sample were determined by measuring the optical density using the NanoDrop 2000c spectrophotometer (Thermo Scientific, Waltham, MA, USA). Subsequently, cDNA synthesis was performed using the High-Capacity iScript cDNA Synthesis Kit (BIO RAD, Hercules, CA, USA). Primer and probe sets were designed using the NCBI Nucleotide tool, and their sequences can be found in Table 3. For qRT-PCR, the samples were analyzed in duplicate using SYBR green dsDNA-intercalating fluorescent dye (TB Green Premix, Takara #RR420L) in a QuantStudio5 Real-Time PCR system (Applied Biosystems). The PCR cycling conditions involved an initial step of 10 min at 95 °C, followed by 10 s at 95 °C and 30 s at 60 °C for 40–45 cycles. To quantify the results, the double delta Ct method was employed, with normalization of the Ct values using the WT Naïve mice values. The gene expression data were further normalized to the 18S housekeeping gene and presented as arbitrary units.

### 4.8. Statistical Analyses

Data are presented as means ± standard error of the mean (SEM), with the number of independent experiments and the statistical analysis used specified in each figure. The Kolmogorov–Smirnov test was used to assess population distribution. When the data did not follow a normal distribution, non-parametric tests (Kruskal–Wallis with post hoc pairwise comparisons using Dunn’s test) were used. When normality was assumed, One-way or Two-way analysis of variance (ANOVA) was performed, depending on the experiment, and followed by a Bonferroni post hoc test. For comparisons between two groups, data were analyzed using Student’s *t*-test when normally distributed, and the Mann–Whitney U test was used if the data did not meet the assumptions for parametric analysis. Statistical analysis and graphical representation were performed using GraphPad Prism 8 software.

## 5. Conclusions

These findings identify TSP-1 and occludin as promising biomarker candidates in the context of TBI, with both associated with long-term outcomes and occludin showing potential for early diagnosis through its ability to differentiate patients with a mild brain injury from those without on CT scans. Moreover, TSP-1 plays a key role in BBB maintenance and post-TBI recovery in our TBI model, with its deficiency leading to worsened neuroinflammation and impaired angiogenic repair, supporting its relevance as a potential therapeutic target. These findings highlight the importance of a dynamic, multimodal approach to biomarker research. However, further studies in larger and multicenter cohorts are necessary to validate the clinical utility of TSP-1 and occludin as possible diagnostic and prognostic tools in TBI management.

## Figures and Tables

**Table 1:** Demographic characteristics of La Paz Hospital cohort.

	Controls	1 day	3 days	7 days
	23	24	24	22
Sex (men/women)	9/14		25/7	
Age (men/women)	58.2 ± 11.2/47.8 ± 16.1	51.8 ± 19.7/62.9 ± 8.2		
Severity (GCS)				
Mild	-	14	14	13
Severe	-	10	10	9
Prognosis (GOSE)				
Favorable	-	11	12	9
Disfavorable	-	13	12	13

**Table 2:** Demographic characteristics of Vall D’Hebron Hospital cohort.

	Controls	CT Normal	CT Pathological
N	24	73	11
Sex (men/women)	11/13	29/44	5/6
Age	53.0 ± 16.5/59.8 ± 19.6	47.4 ± 21.4/64.3 ± 22.5	61.6 ± 23.2/76.3 ± 24.0
TSP-1 levels (ng/mL)	14,015.80 ± 10,245.56	14,570.64 ± 10,114.63	11,178.53 ± 7043.65
Occludin levels (ng/mL)	0.391 ± 0.665	0.512 ± 0.866	0.946 ± 0.920

**Table 3:** Oligonucleotide Primer Sequences for qPCR/PCR.

Gene	Forward primer	Reverse primer
***18s***	5′-CGCCGCTAGAGGTGAAATTCT-3′	5′-CATTCTTGGCAAATGTCTTTCG-3′
***Ccl2***	5′-ACAAGAGGATCACCAGCAGC-3′	5′-GGACCCATTCCTTCTTGGGG-3′
***Nlrp3***	5′-GCCCAAGGAGGAAGAAGAAG-3′	5′-TCCGGTTGGTGCTTAGACTT-3′
***Il1b***	5′-AACCTGCTGGTGTGTGACGTTC-3′	5′-CAGCACGAGGCTTTTTTGTTGT-3′
***Tnfa***	5′-GCCTCTTCTCATTCCTGCTTG-3′	5′-CTGATGAGAGGGAGGCCATT-3′
***Vegf***	5’- CCACGTCAGAGAGCAACATCA-3’	5’-TCATCTCTCCTATGTGCTGGCTTT-3’

## Data Availability

The original contributions presented in this study are included in the article. Further inquiries can be directed to the corresponding author(s). However, individual patient data protected by confidentiality and ethical regulations will not be shared.

## Abbreviations

The following abbreviations are used in this manuscript:

TBI	Traumatic Brain Injury
BBB	Blood–brain barrier
TSP-1	Thrombospondin-1
GCS	Glasgow Coma Scale
CT	Computed Tomography
CHI	Closed Head Injury
AUC	Area Under the Curve
IL1-β	Interleukin-1 beta
TNF-α	Tumor Necrosis Factor alpha
VEGF	Vascular Endothelial Growth Factor
CCL2	Monocyte Chemoattractant Protein-1
NLRP3	NOD-like receptor family, pyrin domain containing 3
